# The Story of the Insane from Year to Year

**Published:** 1902-08-16

**Authors:** 


### THE STORY OF THE INSANE FROM YEAR TO YEAR.

### Fifteenth Annual Series.

### MIDDLESEX COUNTY ASYLUM AT WANDSWORTH.

Here the year opened with 1,415 patients in residence, and by the end of December the number was reduced to 1,314. There were 389 first admissions, 59 readmissions, 170 recoveries, 65 discharged relieved, and 150 deaths. The total number under treatment during the year was 1,846, and the average number resident was 1,401. The recoveries were 42·7 per cent. of the admissions, and the deaths were 10·7 per cent. of the average number resident. Of the year's deaths 56 were from cerebral and spinal diseases, general paralysis alone accounting for 26 of these, consumption was the cause in 26 instances, bronchitis in 4, pneumonia in 1, enteric fever in 2, and enteritis in 4. Of the admissions, 65 owed their insanity to such moral causes as domestic trouble, adverse circumstances, mental anxiety and fright. Intemperance in drink accounts for 30, heredity for 73, previous attacks for 95, and in 125 the cause was unknown. The asylum does not possess sufficient accommodation for the county, and room for 160 patients has to be found elsewhere, and this boarding-out system is the cause of much trouble to the asylum, to the patients, to the patients' friends, and of much additional expense to the ratepayers. Dr. Gardiner Hill very properly lifts up his voice against the practice of having insane inebriates sent from St. Joseph's Reformatory to his asylum, and it is certainly bad enough to have to admit criminals who really belong to the county, without making the asylum a refuge for those who have no claim on it beyond a temporary residence in the neighbourhood. The committee say they continue to receive the most satisfactory testimony as to the kindness and attention shown by the medical officers and staff to the patients. The weekly charge to the Unions is 12s. per head.

### CUMBERLAND AND WESTMORLAND ASYLUM.

At the beginning of the year this asylum contained 665 patients, and there were 664 in residence at the close of the year. There were 132 first admissions, 43 readmissions, 96 discharged recovered, 35 discharged relieved, and 44 died. The recoveries were 54 9 per cent. of the admissions, and the deaths were 6·5 per cent. of the average number resident, the former being decidedly high and the latter low. Turning to the statistics and scanning Table X we notice that 19 of the year's admissions owed their insanity to moral causes, 31 to drink, 48 to hereditary influence, 50 to previous attacks, and in 30 no cause could be ascertained. Of the deaths 16 were caused by cerebral and spinal diseases, only 2 by consumption and 7 by other lung diseases. Dr. Farquharson has some well expressed remarks on the advantages of early treatment in insanity. He shows that in his asylum of the cases sent in where the insanity had been less than a year in duration 52 per cent. recovered, while in those where the year was exceeded the recoveries were only 12 per cent., and these percentages refer to a period of five years. Dr. Farquharson further thinks that more patients could be cured if he had "hospital accommodation" for the treatment of recent and curable cases. We greatly doubt this. Up to the present time it cannot be said to have been proved. He proceeds: "The primary object of an asylum is to promote recovery of those suffering from mental disease." That need not be questioned: but when Dr. Farquharson says that "the more of the insane there are cured the smaller is the burden upon the community in maintaining them," we entirely disagree with him. We have pointed out more than once in these notices that the argument is unsound. Supposing that all the 664 cases in the asylum at the end of the year could be detained till the end of their lives, is anything more certain than that the proportion of the insane throughout the two counties would be materially reduced; and if all the 664 cases could be discharged to-morrow, is it not equally certain that there would be an increase of insanity in the future? By curing 54·9 per cent. Dr. Farquharson is steering a sort of middle course and adding a considerable quota to the burden of the next generation of ratepayers instead of reducing that burden as he thinks. Of course, it must be admitted that Dr. Farquharson must cure as many cases as he possibly can. It is the fallacy of his statement that we quarrel with—not with his high percentage of cures. The average weekly cost per head was 8s. 6d.

